# Neoadjuvant Cemiplimab in Cutaneous Squamous Cell Carcinoma: Complete Primary Tumor Response with Regional Nodal Metastases Case Report

**DOI:** 10.3390/reports9030210

**Published:** 2026-07-03

**Authors:** Seung Hwan Chung, Hussein Ali-Ahmad, Andrew Zwyghuizen, Linda Qu

**Affiliations:** 1 College of Human Medicine, Michigan State University, East Lansing, MI 48824, USA; chungs17@msu.edu; 2 Sparrow Health System, Lansing, MI 48912, USA; hussein.ali-ahmad@umhsparrow.org (H.A.-A.); andrewz@med.umich.edu (A.Z.)

**Keywords:** cutaneous squamous cell carcinoma, cemiplimab, PD-1 inhibition, neoadjuvant immunotherapy, immunotherapy biomarkers

## Abstract

**Background and Clinical Significance:** Cutaneous squamous cell carcinoma (CSCC) is a common non-melanoma skin cancer, and while most cases are curable, a small proportion progresses to locally advanced or metastatic disease. As neoadjuvant immunotherapy with PD-1 inhibitors such as cemiplimab becomes more widely adopted, understanding real-world patterns of response remains essential. **Case Presentation:** We report a case of a man in his 50s with a large, locally advanced CSCC of the left hand in whom neoadjuvant cemiplimab was chosen to reduce tumor burden and preserve hand function when margin-negative resection was unlikely. The patient received four cycles of cemiplimab and demonstrated marked clinical improvement followed by complete pathological response at the primary site upon wide local excision. However, metastatic involvement of the epitrochlear and axillary lymph nodes was identified at surgery despite initial benign imaging. Postoperative PET/CT showed no additional disease, and the patient subsequently underwent axillary dissection and adjuvant cemiplimab with good functional recovery. **Conclusions:** This case highlights the potential for neoadjuvant cemiplimab to achieve substantial local tumor control and functional preservation while emphasizing the need for careful nodal assessment and ongoing surveillance in patients with very-high-risk CSCC. In cases where baseline cross-sectional staging is not performed, pre-existing occult nodal disease cannot be excluded.

## 1. Introduction and Clinical Significance

Cutaneous squamous cell carcinoma (CSCC) is the proliferation of atypical keratinocytes in the skin and is the second most common form (20%) of all non-melanoma skin cancers [1]. The incidence of CSCC has been on the rise over the past 10 years, largely due to the aging population and improved screening and awareness [2,3]. While the majority of cases are effectively managed with localized treatments such as surgery or radiotherapy, a small subset (3~5%) can progress to locally advanced or metastatic disease [4,5]. Metastatic CSCC, though rare, poses a significant clinical challenge due to its aggressive nature and limited treatment options [4,5]. Cemiplimab, a PD-1 inhibitor, has shown promise in advanced CSCC, particularly in reducing tumor burden and improving surgical outcomes.

CSCC are neoplasms characterized by a high mutational burden and alterations in the tumor microenvironment, including dysregulation of immune checkpoint signaling, which enables malignant cells to evade immune detection and destruction [6]. Consequently, they have a greater propensity to respond positively to immune checkpoint inhibitors owing to heightened neoantigen expression [7,8]. Monoclonal antibodies targeting the programmed cell death receptor-1 (PD-1), such as cemiplimab, have exhibited promising efficacy in managing metastatic or locally advanced cutaneous squamous cell carcinoma. These agents enhance the T-cell response and have robust antitumor activity in patients with unresectable CSCC. In a landmark study of 78 patients with advanced CSCC not amenable to surgery or radiation, cemiplimab demonstrated excellent efficacy with a good tolerability profile with few grade 3–4 treatment adverse events [8]. The efficacy of cemiplimab in the first-line setting has led to its evaluation in a neoadjuvant setting for resectable stage II-IV CSCC. Interestingly, about 50% of those patients experienced a pathological complete response with four neoadjuvant cycles, highlighting the significant impact of this drug on the natural history of the disease [9].

In this case report, we describe a patient with very-high-risk locally advanced cutaneous squamous cell carcinoma of the left hand who was treated with four cycles of neoadjuvant cemiplimab in accordance with NCCN-guided risk stratification and multidisciplinary treatment planning. Neoadjuvant therapy was selected to reduce tumor burden and preserve hand function when margin-negative resection was considered unlikely without substantial morbidity. Following treatment, the patient achieved complete pathological response at the primary tumor site, while regional nodal metastases were identified at surgery despite benign-appearing baseline imaging findings. This case highlights the potential role of neoadjuvant PD-1 blockade in achieving local tumor control while emphasizing the importance of careful nodal evaluation and surveillance in very-high-risk CSCC.

## 2. Case Presentation

The patient is a man in his mid-50s who initially presented with a small, slow-growing, ulcerated lesion on the dorsum of the left hand in June 2020 (Figure 1). At the time, he was recommended dermatology evaluation but was lost to follow-up. He re-presented in April 2024 with significant interval growth of the lesion, which now occupied the majority of the dorsum of the left hand and caused significant functional impairment (Figure 2, Table 1). The patient’s past medical history was otherwise non-contributory.

Incisional skin biopsy of the left-hand lesion was performed, with pathology showing invasive, well-differentiated squamous cell carcinoma with positive margins. Unfortunately, additional pathologic features such as depth of invasion and perineural invasion were not reported. The primary carcinoma was categorized as very-high risk CSCC based on NCCN risk stratification due to its large size (>4 cm) [10]. The patient was noted to have a palpable left epitrochlear lymph node without palpable left axillary lymphadenopathy. Further workup with nodal basin ultrasound was ordered, which revealed benign-appearing, morphologically normal left epitrochlear (Figure 3a,b) and left axillary nodes (Figure 4a–c). MRI of the left arm was also completed, which revealed a mildly enlarged, 1.5 cm, medial epicondylar lymph node, with axillary nodes that were nonpathologic by size and morphologic features with benign fatty hila. His case was next discussed at multidisciplinary tumor board, and, given the absence of radiographically concerning features on both US and MRI, and absence of systemic symptoms worrisome for metastatic disease, no nodal biopsy or systemic staging was pursued. He was therefore staged as clinical stage II, cT2 cN0 Mx, given (a) the low likelihood of achieving margin negative resection, given the extensive local involvement, (b) to mitigate the need for hand amputation to achieve margin negative resection, and (c) the patient’s young age and excellent performance status, rendering preservation of function of utmost importance, consensus recommendation was to proceed with neoadjuvant cemiplimab.

The patient completed four cycles of neoadjuvant cemiplimab between May and July 2024. After two cycles, marked improvement in the primary lesion was already demonstrated (Figure 5).

Following four cycles of treatment, the patient underwent wide local excision of the left hand CSCC with excision of the left epitrochlear lymph node and left axillary sentinel lymph node biopsy. Reconstruction was initiated with a first stage synthetic dermal matrix (Novosorb BTM bi-layer) for interval coverage pending clear margins. Final surgical pathology demonstrated no residual carcinoma in the wide local excision specimen, consistent with pathologic complete response, but with metastatic squamous cell carcinoma in the epitrochlear lymph node and 2/2 left axillary sentinel lymph nodes positive for metastatic CSCC. Because no residual viable carcinoma was identified in the post-treatment excision specimen, final depth of invasion, margin status, perineural invasion, lymphovascular invasion, and other histologic features are not applicable. Postoperative staging PET/CT revealed mild uptake in the left axilla, compatible with postsurgical change, without other evidence of metastatic disease (Figure 6). After multidisciplinary discussion, the patient underwent completion left axillary lymph node dissection. This procedure was coordinated with second stage split-thickness skin grafting to complete his reconstruction. Final pathology revealed no further metastatic involvement among the eight additional nodes dissected.

The patient was ultimately staged pathologic stage IV, ypT0 ypN2b M0. Adjuvant radiation to the axillary nodal basin was considered but was deferred given the compounded risk of lymphedema and in the setting of his skin-grafted left hand. Adjuvant radiation to the left hand was deferred given the patient’s pathologic complete response. Given his high-risk disease, however, and extrapolating from cemiplimab use in the metastatic setting, the patient was restarted on adjuvant cemiplimab to complete 1 year of therapy. He tolerated therapy well without side effects and PET surveillance during adjuvant treatment revealed no evidence of recurrent or metastatic disease. He received his final cycle of cemiplimab on December 2025, approximately 16 months after surgery, and post-treatment PET showed no evidence of disease. At last clinical follow-up in June 2026 (26 months after diagnosis), he continued to do well with no clinical evidence of recurrence. The patient overall recovered extremely well with complete graft take. He returned to full use of the hand with excellent range of motion and hand function (Figure 7).

## 3. Discussion

This case illustrates both the potential benefits and the limitations of neoadjuvant cemiplimab in the management of very-high-risk CSCC. Cemiplimab has demonstrated significant potential in enhancing the immune system’s ability to recognize and target tumor antigens, an approach that is particularly relevant in CSCC, which exhibits a high tumor mutational burden due to ultraviolet-induced DNA damage [6]. Studies have shown that CSCC has a median tumor mutational burden of approximately 45 mutations per megabase, three times higher than that of skin melanoma [11]. This elevated mutational load is associated with increased immunogenicity, potentially improving the efficacy of PD-1 blockade. In this patient, whose diagnosis of a large hand CSCC would likely have been unresectable without amputation, the use of neoadjuvant cemiplimab was associated with complete clinical regression and allowed for functional preservation. The complete pathological response observed at the primary tumor site is consistent with prior studies demonstrating substantial activity of neoadjuvant PD-1 inhibition in resectable CSCC [9].

Importantly, however, metastatic involvement of the epitrochlear and axillary lymph nodes was identified at surgery despite benign-appearing baseline ultrasound and MRI findings. Baseline imaging was obtained in accordance with NCCN guidelines for very-high-risk CSCC, which recommend MRI with and without contrast or CT and/or ultrasound, with biopsy reserved for abnormal lymphadenopathy seen on imaging. Per NCCN, PET imaging is reserved for cases of biopsy-proven nodal disease. This baseline staging strategy deserves careful consideration. In this case, management was guided by NCCN risk stratification, multidisciplinary review, clinical nodal examination, and regional ultrasound and MRI findings [10]. Because no radiographically suspicious nodal morphology or systemic symptoms were present, nodal biopsy and baseline cross-sectional systemic staging with PET/CT was not pursued prior to neoadjuvant cemiplimab. However, given the very-high-risk nature of the primary tumor and the presence of a palpable epitrochlear node, additional nodal evaluation, including ultrasound-guided biopsy or cross-sectional staging, could have also been considered. As a result, it remains uncertain whether nodal metastases developed during therapy or were already present but undetected at baseline. The findings can therefore be interpreted as discordant response characterized by complete pathological response of the primary lesion with persistent or subsequently detected regional nodal metastases.

Despite this discordance, the patient ultimately recovered well and completed treatment with full functional preservation of his left hand and has no evidence of disease more than 2 years after initial diagnosis. The decision to continue adjuvant cemiplimab was made in light of his high-risk disease, before results of the adjuvant C-POST Trial were published. In this trial, adjuvant cemiplimab improved disease-free survival over placebo for patients with high-risk CSCC (defined as at least three involved nodes, extracapsular extension with largest node ≥ 20 mm, in-transit metastases, T4 lesion, PNI, or locally recurrent tumor). The estimated 24-month disease-free survival was 87.1% with cemiplimab versus 64.1% with placebo, with a hazard ratio for disease recurrence or death of 0.32 (95% CI, 0.20–0.51; *p* < 0.001) [12]. While trial patients were also treated with postoperative radiotherapy, the decision was made to omit adjuvant radiation in this patient to mitigate the risk of lymphedema, especially following axillary lymph node dissection. Nevertheless, the clinical benefit of adjuvant cemiplimab in this case has so far been sustained.

An interesting component of this case that is worth discussing is the differing degree of PD-L1 expression in the primary tumor compared to the positive lymph nodes. PD-L1 semi-quantitative immunohistochemistry revealed 2% membranous positivity (combined positive score [CPS]) in the primary lesion (pre-cemiplimab) and 10% membranous positivity in the positive lymph nodes (post-cemiplimab). We hypothesize that even occult nodal disease should have responded to neoadjuvant cemiplimab, yet nodal metastases were identified at surgery, underscoring the potential limited predictive utility of PD-L1 status alone. Indeed, the relevance of the proportion of PD-L1 expression in predicting the response to PD-1 inhibitors remains controversial and differs for different cancers. For CSCC, a phase II trial showed that patients with PD-L1-positive tumors have a higher rate of pathological complete response compared to those with PD-L1-negative tumors [9]. A study of another PD-1 inhibitor, nivolumab, in resected esophageal and gastroesophageal junction cancers showed significantly improved disease-free survival regardless of PD-L1 expression [13]. Studies with nivolumab in non-small cell lung cancer, on the other hand, showed conflicting results based on histological subtype [14,15]. Additionally, the location of the tumor may matter as well. A retrospective, observational, multicenter study performed in Italy found that primary tumor site was significantly associated with response to cemiplimab, with tumors arising on the head and neck showing a better response rate when compared to other anatomical sites (63.8% versus 37.8%, *p* = 0.007) [16]. These findings raise the possibility that disease site or histology, rather than the percentage of PD-L1 expression, could serve as a predictor of response to immunotherapy. However, further research is needed to confirm this hypothesis. Furthermore, other studies have found that in addition to PD-L1, other tumor biomarkers including IFN-γ signaling, genomic profiling, and JAK mutations can contribute to tumor cell’s response to immunotherapy [11,17,18]. These analyses were not performed in this patient. Future studies incorporating comprehensive molecular profiling may help clarify mechanisms underlying heterogeneous responses to immunotherapy in CSCC, particularly in very-high-risk patients.

## 4. Conclusions

In summary, this case demonstrates complete pathological response of a very-high-risk locally advanced CSCC of the hand following neoadjuvant cemiplimab, while regional nodal metastases were identified at surgery despite clinically negative lymphadenopathy at diagnosis. Current guidelines do not routinely recommend baseline systemic staging or tissue confirmation in the absence of radiographically abnormal lymph nodes, highlighting the need for multidisciplinary evaluation and management of high-risk patients on a case-by-case basis. In this report, it remains uncertain whether nodal disease developed during therapy or was present but undetected before treatment. The case highlights the potential role of neoadjuvant cemiplimab in facilitating local tumor control and functional preservation, while emphasizing the importance of careful nodal evaluation and ongoing surveillance in patients with very-high-risk CSCC. Further investigation is needed to define optimal staging strategies, adjuvant management, and predictors of response in this population.

## Figures and Tables

**Figure 1:**
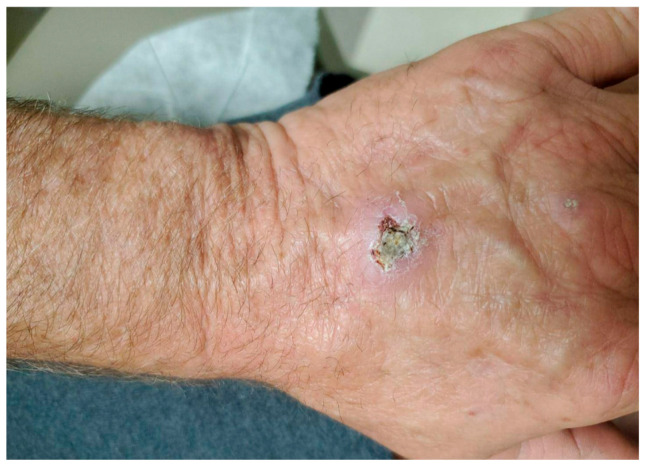
Patient’s initial left hand lesion in June 2020.

**Figure 2:**
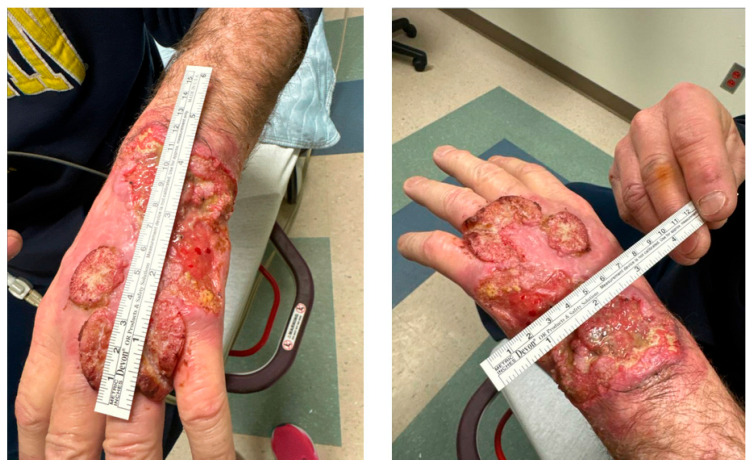
Patient evaluated 4 years later with a progressive, ulcerated left hand lesion, measuring 12 cm from the wrist extending to the metacarpophalangeal joints with multiple areas of fungating tissue growth.

**Figure 3:**
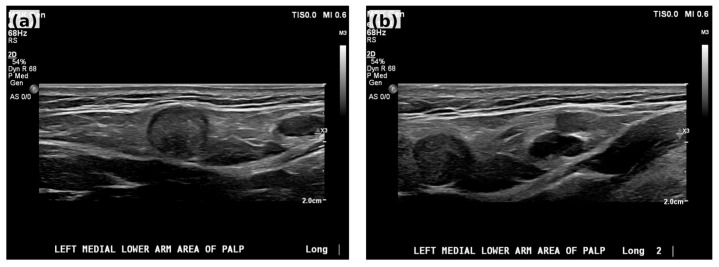
Ultrasound of two benign-appearing epitrochlear lymph nodes: (**a**) 3 × 18 × 9 mm; (**b**) 10 × 9 × 4 mm.

**Figure 4:**
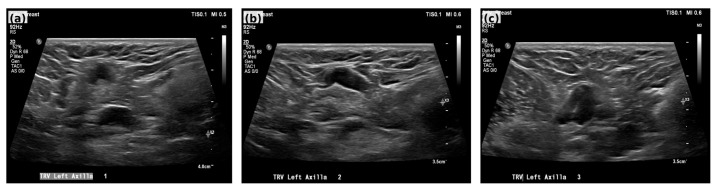
(**a**–**c**) Ultrasound of three morphologically normal left axillary lymph nodes.

**Figure 5:**
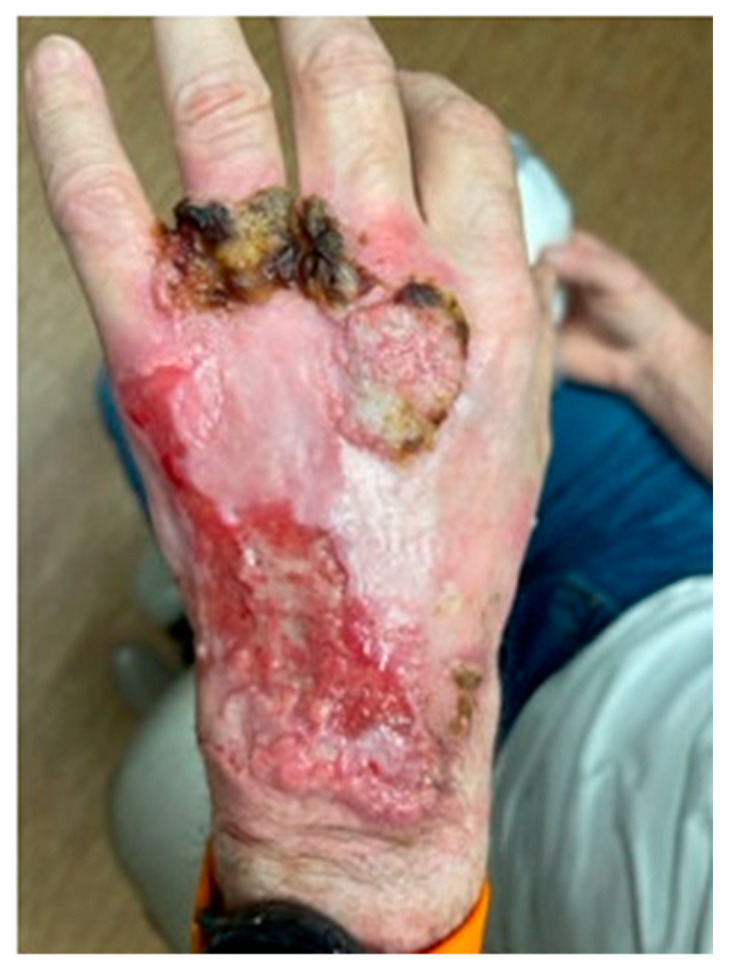
Left hand lesion after 2 cycles of neoadjuvant cemiplimab.

**Figure 6:**
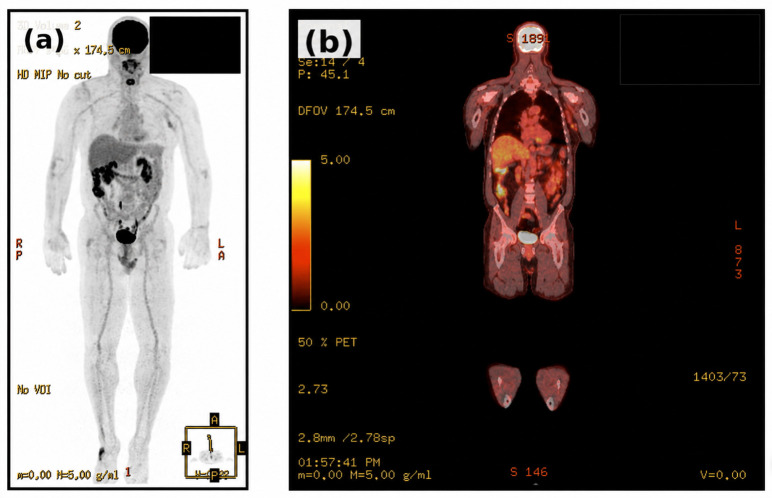
Postoperative PET/CT imaging obtained after wide local excision and sentinel lymph node biopsy. (**a**) Maximum intensity projection image demonstrating physiologic FDG distribution without evidence of distant metastatic disease. (**b**) Coronal fused PET/CT image showing mild FDG uptake within the left axilla, interpreted as postsurgical inflammatory change rather than residual metastatic disease.

**Figure 7:**
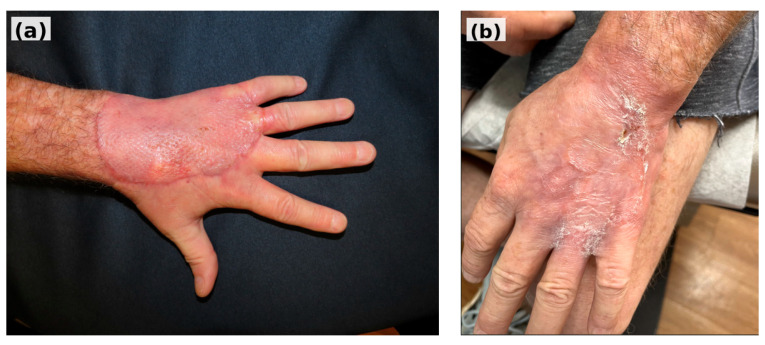
(**a**) Left hand post-grafting. (**b**) Left hand post-grafting at 4 weeks into adjuvant cemiplimab.

**Table 1:** Clinical Timeline.

Date	Clinical Event
June 2020	Initial ulcerated lesion noted on the dorsum of the left hand; dermatology evaluation recommended, but patient lost to follow-up.
April 2024	Re-presentation with an approximately 12 cm ulcerated, fungating lesion involving the dorsum of the left hand and causing functional impairment. Incisional biopsy confirmed invasive well-differentiated CSCC with positive margins. Palpable left epitrochlear node noted; no palpable left axillary lymphadenopathy. Ultrasound of epitrochlear and axillary nodal basins demonstrated benign-appearing, morphologically normal lymph nodes. MRI of left arm demonstrated mildly enlarged, 1.5 cm medial epicondylar lymph node and nonpathologic, benign-appearing axillary nodes. No biopsy pursued.
May 2024	Case discussed at multidisciplinary tumor board with consensus recommendation to proceed with neoadjuvant cemiplimab.
May–July 2024	Four cycles of neoadjuvant cemiplimab administered.
August 2024	Wide local excision of the left-hand CSCC, excision of the palpable left upper arm/epitrochlear lymph node and left axillary sentinel lymph node biopsy performed. Pathology demonstrated pathologic complete response in left hand; metastatic CSCC identified in left epitrochlear lymph node; metastatic CSCC identified in 2/2 left axillary sentinel lymph nodes.
September 2024	PET/CT demonstrated mild left axillary uptake interpreted as postsurgical change and no evidence of distant metastatic disease.
September 2024	Completion left axillary lymph node dissection and split-thickness skin grafting performed; 8 additional lymph nodes were negative for metastatic involvement.
November 2024	Adjuvant cemiplimab started.
January 2025	PET/CT showed no evidence of recurrent or metastatic disease.
July 2025	PET/CT showed no evidence of recurrent or metastatic disease.
December 2025	Final cycle of adjuvant cemiplimab administered. Post-treatment PET/CT showed no evidence of recurrent or metastatic disease.
June 2026	At last follow-up, no clinical evidence of recurrent or metastatic disease. Plan for surveillance PET annually (next due in December 2026).

## Data Availability

The original contributions presented in this study are included in the article. Further inquiries can be directed to the corresponding author.
